# Adults With Type 1 Diabetes Are Less Physically Active Than Adults Without Diabetes: A Systematic Review and Meta‐Analysis

**DOI:** 10.1002/dmrr.70250

**Published:** 2026-11-06

**Authors:** Antonio García‐Hermoso, Jacinto Muñoz‐Pardeza, Ignacio Hormazábal‐Aguayo, Yasmin Ezzatvar

**Affiliations:** ^1^ Navarrabiomed University Hospital of Navarra Public University of Navarra (UPNA) Navarre Health Research Institute (IdiSNA) Pamplona Spain; ^2^ Vicerrectoría de Investigación y Postgrado Universidad de La Serena La Serena Chile; ^3^ Department of Nursing Lifestyle Factors With Impact on Ageing and Overall Health (LAH) Research Group University of València Valencia Spain; ^4^ Vicerrectoría de Investigación y Postgrado Universidad de Los Lagos Osorno Chile

**Keywords:** accelerometry, exercise, insulin‐dependent diabetes mellitus, movement behaviours

## Abstract

**Aims:**

Physical activity is central to the management of type 1 diabetes, yet diabetes‐specific barriers may reduce habitual activity. We systematically reviewed and meta‐analysed studies comparing physical activity and sedentary behaviour in adults with type 1 diabetes and adults without diabetes.

**Material and Methods:**

MEDLINE, Embase, and SPORTDiscus were searched from inception to 24 July 2026. Random‐effects models were used to pool standardised mean differences for continuous outcomes and odds ratios (OR) for meeting physical activity recommendations or study‐defined active criteria.

**Results:**

Sixteen publications representing 14 independent cohorts and 3335 participants were included (54.2% females). Adults with type 1 diabetes had lower total physical activity than adults without diabetes (12 studies; Hedges *g* = −0.13; 95% confidence interval, −0.22 to −0.05; *p* = 0.001), with no detected heterogeneity. They also had lower estimated odds of meeting a physical activity criterion, although the association was not statistically significant (4 studies; OR = 0.77; 95% confidence interval, 0.58–1.02; *p* = 0.066). Mean age, diabetes duration and glycated haemoglobin were not associated with between‐group differences in total physical activity. Data were insufficient to pool sedentary behaviour, steps or intensity‐specific outcomes.

**Conclusions:**

Adults with type 1 diabetes engage in slightly less total physical activity than adults without diabetes, although evidence regarding adherence to physical activity recommendations remains inconclusive. The absence of population‐based studies limits the generalisability of these findings and highlights the need for representative studies using standardised device‐based assessments.

## 1 Introduction

Physical activity is a key component of the management of type 1 diabetes and is recommended to improve general health, cardiovascular fitness, psychological well‐being, and long‐term cardiometabolic risk [1]. Current guidelines recommend that most adults with diabetes accumulate at least 150 minutes of moderate‐to‐vigorous aerobic activity per week, distributed across at least 3 days, together with 2–3 weekly sessions of resistance exercise and a reduction in prolonged sedentary time [2]. In adults with type 1 diabetes, exercise training has been associated with improvements in cardiorespiratory fitness, lipid profile, vascular function, body composition, and insulin requirements [3]. However, its effect on long‐term glycaemic control remains less consistent, with previous systematic reviews reporting variable or modest changes in HbA1c [3, 4]. These broader health benefits remain particularly relevant because adults with type 1 diabetes continue to experience an increased burden of cardiovascular disease despite improvements in insulin therapy and diabetes technology [5].

Engaging in regular physical activity can nevertheless be challenging for people with type 1 diabetes. The glycaemic response to physical activity varies according to its intensity, duration, timing, and accumulated volume, as well as pre‐activity glucose concentrations, circulating insulin, carbohydrate intake, and recent activity patterns [1]. As a result, even routine or leisure‐time activities may require additional glucose monitoring and adjustments to insulin delivery or carbohydrate intake. Fear of hypoglycemia, concern about glycaemic variability, insufficient knowledge of activity management, and the additional self‐management burden have consistently been identified as barriers to an active lifestyle [1, 6, 7]. These barriers are clinically relevant because persistently low physical activity has been associated with higher all‐cause mortality in adults with type 1 diabetes, whereas higher activity levels may be associated with lower cardiovascular risk [8, 9]. Sedentary behaviour may also confer additional risk, for example, in the prospective FinnDiane study, occupational sitting for at least 6 hours per day was associated with a 55% higher risk of cardiovascular events, independently of leisure‐time physical activity [10].

Evidence from younger populations suggests that differences in physical activity may emerge early in type 1 diabetes. A previous systematic review and meta‐analysis found that children and adolescents with type 1 diabetes were less active, accumulated less moderate‐to‐vigorous physical activity, spent more time sedentary, and were less likely to meet physical activity recommendations than peers without diabetes [11]. However, whether these differences persist into adulthood remains uncertain. A recent meta‐analysis also reported lower cardiorespiratory fitness in adults with type 1 diabetes [12], although fitness reflects physiological capacity rather than habitual physical activity. Therefore, this systematic review and meta‐analysis aimed to compare physical activity and sedentary behaviour between adults with type 1 diabetes and healthy adults without diabetes and to explore whether study‐level age, diabetes duration, and HbA1c were associated with between‐group differences in total physical activity.

## 2 Methods

This systematic review and meta‐analysis was conducted and reported in accordance with the Preferred Reporting Items for Systematic Reviews and Meta‐Analyses (PRISMA) 2020 statement and was registered in PROSPERO (CRD420261460555).

### 2.1 Eligibility Criteria

We included original studies comparing adults aged ≥ 18 years with type 1 diabetes with adults without diabetes. Eligible studies reported at least one measure of physical activity or sedentary behaviour, including total physical activity, moderate‐to‐vigorous physical activity, moderate physical activity, vigorous physical activity, light physical activity, steps per day, sedentary time, sitting time, screen time, or meeting physical activity guidelines. Physical activity and sedentary behaviour could be assessed using device‐based methods, such as accelerometers, pedometers or actigraphy, or validated self‐report instruments. Observational studies and baseline data from intervention studies were eligible, provided that a healthy control group was included. Studies with mixed diabetes populations were included only when data for adults with type 1 diabetes were reported separately. Studies in which participants were selected on the basis of being physically active, athletic status, or engagement in structured exercise were excluded from the primary analysis. Studies in which controls were explicitly matched to participants with type 1 diabetes on physical activity, leisure‐time physical activity, fitness, or activity history were also excluded from the primary analysis and retained, when appropriate, for sensitivity analyses. Studies conducted during COVID‐19 lockdown or restriction periods were excluded from the primary analysis because physical activity and sedentary behaviour were likely influenced by pandemic‐related restrictions.

### 2.2 Information Sources

We searched MEDLINE via PubMed, Embase, and SPORTDiscus from database inception to 24 July 2026. The reference lists of all included studies and relevant systematic reviews were screened to identify additional potentially eligible publications. No restrictions were applied according to publication year. Searches were restricted to publications in English and Spanish. When necessary, study authors were contacted to request missing or insufficiently reported information.

### 2.3 Search Strategy

The search strategy combined controlled vocabulary terms and free‐text terms relating to type 1 diabetes, physical activity, and sedentary behaviour. Search terms included variations of ‘type 1 diabetes’, ‘type I diabetes’, ‘T1D’, and ‘insulin‐dependent diabetes’, together with terms such as ‘physical activity’, ‘exercise’, ‘moderate‐to‐vigorous physical activity’, ‘accelerometer’, ‘pedometer’, ‘steps’, ‘sedentary behaviour’, ‘sitting time’, and ‘screen time’. Comparator‐related terms were not required in the main search because their inclusion could have reduced sensitivity; the presence of a healthy control group was assessed during study selection. The complete search strategies for all databases are provided in Supporting Information S1: Table 1.

### 2.4 Selection Process

After duplicate removal, 2 reviewers (AGH and YE) independently screened the titles and abstracts of all retrieved records against the prespecified eligibility criteria. Publications considered potentially eligible by either reviewer underwent full‐text assessment. The same reviewers independently assessed the full texts and recorded the primary reason for exclusion of ineligible reports. Disagreements at either stage were resolved through discussion and consensus; when consensus could not be reached, a third reviewer (JMP) adjudicated.

Multiple publications originating from the same cohort were linked during the selection process. Such publications were eligible when they reported different assessment periods, measurement methods, or outcome‐specific analyses. However, they were not regarded as independent cohorts when describing the overall evidence base, and overlapping participants were not counted more than once within the same meta‐analysis.

### 2.5 Data Collection Process

Data were extracted using a standardized form by two reviewers, with disagreements resolved by consensus or a third reviewer. Information was obtained from articles, Supporting Information S1: tables, and figures; graphical data were digitized when necessary, and authors were contacted for missing information. Studies from the same cohort were collated, and one effect estimate per outcome was selected using a prespecified hierarchy to avoid double‐counting, prioritising the broadest total physical activity measure and criteria most closely aligned with established recommendations. Device‐measured moderate‐to‐vigorous physical activity was analysed separately.

### 2.6 Data Items

We extracted study design and setting, sample size, matching procedures, participant characteristics, and relevant clinical variables, including age, sex, diabetes duration, HbA1c, and insulin treatment. For physical activity and sedentary behaviour, we recorded the assessment method, instrument or device, measurement protocol, units, and outcome definitions. Outcomes included total and intensity‐specific physical activity, steps, sedentary time, and meeting a physical activity recommendation or study‐defined active criterion.

For continuous outcomes, sample sizes and summary statistics required to calculate standardized mean differences were extracted; for dichotomous outcomes, events and total participants in each group were recorded. Total physical activity and meeting a physical activity criterion were the primary outcomes, whereas device‐measured moderate‐to‐vigorous physical activity was analysed separately. Mean age, diabetes duration, and HbA1c were collected for exploratory meta‐regression, together with information required for risk‐of‐bias assessment.

### 2.7 Study Risk of Bias Assessment

Risk of bias was assessed using design‐specific Joanna Briggs Institute critical appraisal tools [13]. Cross‐sectional studies and baseline comparisons from intervention studies were evaluated using the JBI Critical Appraisal Checklist for Analytical Cross‐Sectional Studies, whereas case‐control studies were evaluated using the JBI Critical Appraisal Checklist for Case‐Control Studies.

The assessments considered participant selection, comparability of groups, validity and reliability of diabetes and physical activity measurements, identification and management of confounding factors, consistency of outcome measurement, appropriateness of matching procedures, and suitability of the statistical analyses. Two reviewers (AGH and YE) independently assessed each study, rating each item as yes, no, unclear, or not applicable. Disagreements were resolved by consensus or consultation with a third reviewer (JMP).

### 2.8 Effect Measures

For continuous physical activity outcomes, between‐group differences were expressed as standardized mean differences using Hedges *g*, which corrects for small‐sample bias. Effect sizes were calculated as the mean value in participants with type 1 diabetes minus the mean value in controls without diabetes. Thus, negative values indicated lower physical activity among participants with type 1 diabetes, whereas positive values indicated higher physical activity among participants with type 1 diabetes.

For dichotomous outcomes evaluating whether participants met physical activity recommendations or a study‐defined active physical activity criterion, effect estimates were expressed as odds ratios (ORs). An OR of less than 1 indicated lower odds of meeting the physical activity criterion among participants with type 1 diabetes.

When continuous outcomes were reported as medians and interquartile ranges, means and standard deviations were estimated using the methods of Luo et al. [14], respectively. HbA1c values reported in International Federation of Clinical Chemistry units were converted to National Glycohemoglobin Standardisation Programme percentages.

### 2.9 Synthesis Methods

Separate meta‐analyses were conducted for total physical activity and for meeting a physical activity recommendation or study‐defined active criterion. Meta‐analysis was not feasible for the remaining outcomes because too few studies reported comparable data or because outcome definitions and measurement methods were too heterogeneous. Effect estimates were pooled using random‐effects models, with restricted maximum likelihood estimation of between‐study variance.

Statistical heterogeneity was assessed using Cochran Q, the *I* ^2^ statistic, and the between‐study variance estimate (*τ* ^2^). Forest plots were used to present individual and pooled effect estimates with 95% CIs. The robustness of the pooled estimates was examined using alternative models based on restricted maximum likelihood with the Hartung‐Knapp adjustment, Paule‐Mandel estimation, DerSimonian‐Laird estimation, and a common‐effect inverse‐variance model. Leave‐one‐out analyses were performed by sequentially excluding each study and recalculating the pooled estimate.

Exploratory random‐effects meta‐regressions were conducted for total physical activity to evaluate whether the between‐group effect size was associated with mean HbA1c, mean diabetes duration, and mean age. Each moderator was evaluated in a separate univariable model using restricted maximum likelihood estimation with Hartung‐Knapp inference. Regression coefficients were expressed per 1‐percentage‐point increase in HbA1c, per 10‐year increase in diabetes duration, and per 10‐year increase in age.

All statistical tests were two‐sided, and *p* < 0.05 was considered statistically significant. Analyses were performed using R (R Foundation for Statistical Computing) with the *metafor* package in RStudio version 2024.12.1 + 563 (Posit Software, PBC).

### 2.10 Reporting Bias Assessment

For the total physical activity meta‐analysis, potential reporting bias and small‐study effects were assessed by visual inspection of a funnel plot. Funnel plot asymmetry was also evaluated using the Begg rank correlation test and an Egger‐type regression test with the effect‐size standard error as the predictor.

Formal assessment of reporting bias was not conducted for the analysis of meeting a physical activity criterion because only 4 studies were available, which was considered insufficient for reliable funnel plot interpretation or statistical tests of asymmetry [15].

## 3 Results

### 3.1 Study Selection

The database searches identified 4916 records: 2231 from PubMed, 2593 from Embase, and 92 from SPORTDiscus. After removal of 1126 duplicate records, 3790 records underwent title and abstract screening, of which 3731 were excluded. Fifty‐nine reports were assessed for eligibility in full text, of which 43 were excluded. Sixteen publications representing 14 distinct cohorts were included in the systematic review and quantitative synthesis [16, 17, 18, 19, 20, 21, 22, 23, 24, 25, 26, 27, 28, 29, 30, 31]. The study‐selection process is shown in Figure 1, and detailed reasons for exclusion are provided in Supporting Information S1: Table 2.

**FIGURE 1:**
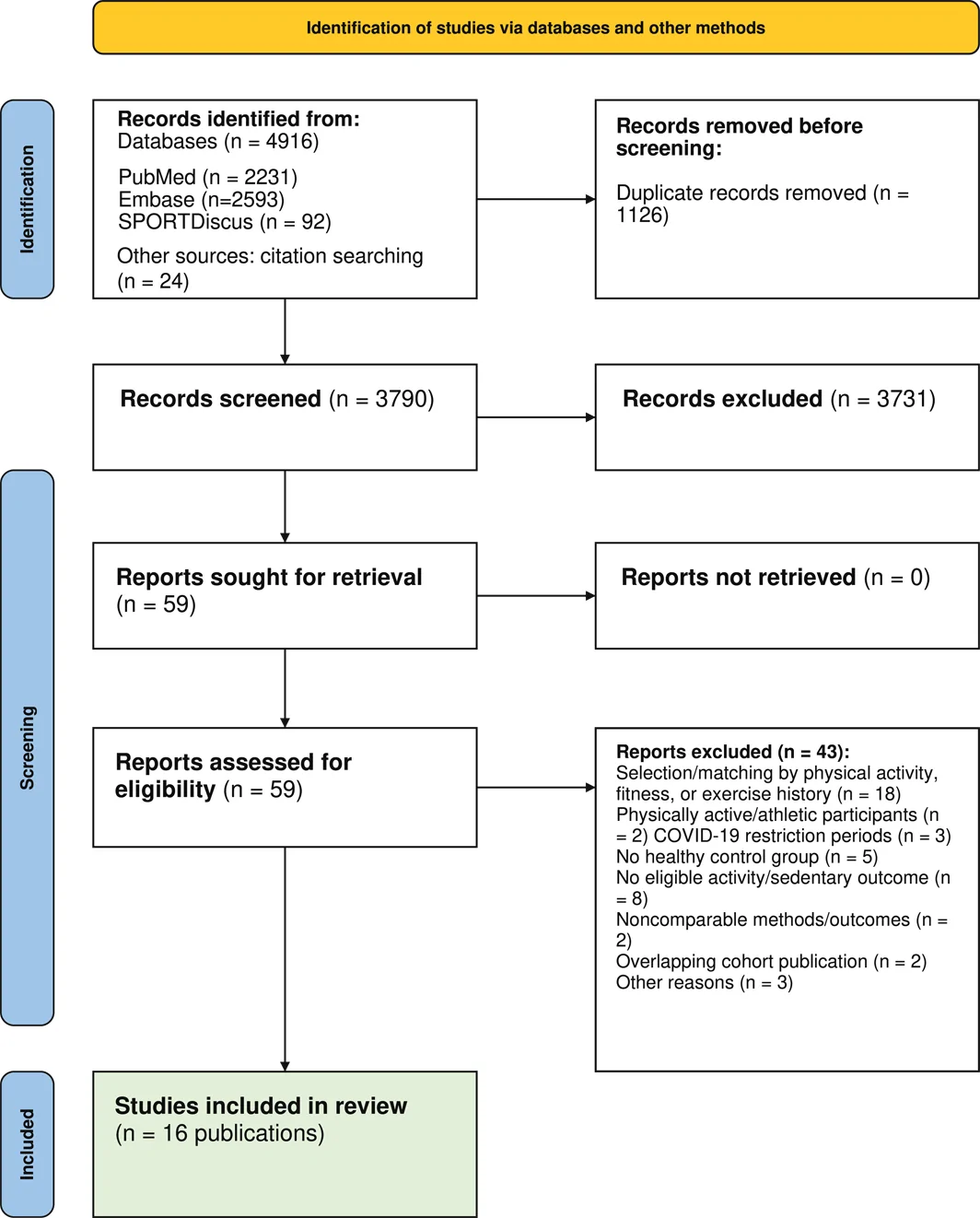
PRISMA flow diagram of study selection.

### 3.2 Study Characteristics

Sixteen eligible publications, published between 1992 and 2026, were included in the quantitative synthesis [16, 17, 18, 19, 20, 21, 22, 23, 24, 25, 26, 27, 28, 29, 30, 31]. Together, they represented 14 distinct cohorts comprising 3335 participants, of whom 1808 (54.2%) were women. None of the included studies used population‐based sampling. Participants were predominantly recruited from clinical settings, convenience samples, or volunteer populations. These publications represented 14 distinct cohorts because Alman et al. [18], Keshawarz et al. [21], and Driscoll et al. [29] analysed data from the Coronary Artery Calcification in Type 1 Diabetes cohort at different assessment periods or for different outcome‐specific analyses. The studies were conducted in North America, Europe, and Australia and were predominantly cross‐sectional observational comparisons. Matson et al. [22] contributed baseline data from an exercise intervention feasibility study. Individual samples ranged from 30 participants in Roberts et al. [23] to 1177 participants in Alman et al. [18].

Physical activity assessment methods varied across studies. Four studies used device‐based measures: Chantelau and Wirth [16] used a portable waist‐worn motion metre; Brazeau et al. [17] used a multisensor armband; and Keshawarz et al. [21] and Matson et al. [22] used waist‐ or hip‐worn ActiGraph accelerometers. The remaining studies used validated questionnaires or structured physical activity histories [18, 19, 20, 23, 24, 25, 26, 27, 28, 29, 30, 31]. Self‐reported instruments included variants of the International Physical Activity Questionnaire [19, 24, 27, 30], the Short Questionnaire to Assess Health‐Enhancing Physical Activity [28], the Modifiable Activity Questionnaire [18, 29], and study‐specific measures of leisure‐time activity, weekly exercise, or adherence to physical activity recommendations [20, 23, 25, 26, 31]. Consequently, reported outcomes included total activity scores, physical activity energy expenditure, metabolic equivalent minutes or hours per week, activity sessions per week, accelerometer‐measured moderate‐to‐vigorous physical activity, and categorical indicators of active or guideline‐adherent behaviour. Sorola et al. [31] assessed self‐reported weekly physical activity according to intensity, frequency, duration, and total hours per week.

Participants were generally young to middle‐aged adults. Among studies with compatible summary data, the mean or estimated mean age of the type 1 diabetes groups ranged from approximately 30 years in Chantelau and Wirth [16] to 51 years in Driscoll et al. [29]. Diabetes duration varied substantially: Matson et al. [22] enroled adults diagnosed with type 1 diabetes within the preceding 3 months, whereas several studies included participants with longstanding type 1 diabetes, with mean diabetes duration exceeding 30 years in some cohorts [20, 21, 28, 29].

Detailed study‐level characteristics are presented in Table 1.

**TABLE 1:** Characteristics of the included studies and physical activity assessment.

Study, country	Design or cohort	Participants, no. T1D/control	Women, no. (%) T1D/control	Age, *y*, T1D/control	Diabetes duration, *y*	HbA1c, %	Physical activity assessment and outcome	Synthesis
Alman et al. [18], US	Cross‐sectional baseline analysis of the CACTI cohort	546/631	310 (56.8)/319 (50.6)	37.0 (9.1)/39.0 (9.1)	23.7 (9.0)	8.0 (95% CI, 7.9–8.1)	Modifiable activity questionnaire; AHA poor, intermediate, or ideal PA category based on moderate and vigorous PA, min/wk	Meeting/active PA
Brandt et al. [28], Denmark	Cross‐sectional study with sex‐ and age‐matched controls	111/37	66 (59.5)/22 (59.5)	42.9 (95% CI, 39.5–46.3)/43.3 (95% CI, 35.2–51.4)	32.2 (14.2)	7.4 (95% CI, 7.2–7.6)	SQUASH; overall activity score and duration of light‐, moderate‐, and vigorous‐intensity PA	Total PA
Brazeau et al. [17], Canada	Cross‐sectional comparison	75/75	35 (46.7)/37 (49.3)	43.5 (10.5)/40.1 (12.8)	23.4 (12.0)	7.56 (1.25)	SenseWear pro 3 armband; physical activity level as the ratio of total to resting energy expenditure and active‐lifestyle threshold of PAL ≥ 1.7	Total PA; meeting/active PA
Chantelau and Wirth [16], Germany	Matched case‐control study	34/34^a^	11 (32.4)/11 (32.4)^a^	30.4/29.6^a^	Range, 4–26	6.92^a^	Portable motion metre worn for 7 consecutive days; habitual PA, arbitrary activity units/wk	Total PA
Driscoll et al. [29], US	Cross‐sectional analysis of CACTI visit 4	414/488	229 (55.3)/247 (50.6)	51.2 (8.9)/54.8 (8.7)	37.5 (8.6)	7.8 (1.1)	Modifiable activity questionnaire; moderate‐equivalent PA and moderate‐to‐vigorous PA, min/wk	Total PA
Falkowski et al. [19], Poland	Cross‐sectional study	90/22	47 (52.2)/17 (77.3)	35.5 (IQR, 29–42)/38.5 (IQR, 30–45)	18 (IQR, 13–26)	8.2 (IQR, 7.3–9.0)	IPAQ‐SF; total PA score	Total PA
Keshawarz et al. [21], US	Cross‐sectional accelerometry substudy of CACTI	44/77	25 (56.8)/40 (52.0)	49 (9)/56 (8)	36 (8)	7.7 (1.4)	ActiGraph wGT3X‐BT triaxial accelerometer; total MVPA, time accumulated in MVPA bouts, and number of MVPA bouts per week	Objective MVPA
Matson et al. [22], UK	Baseline comparison from the EXTOD feasibility study	50/65	18 (36.0)/46 (70.8)	33 (10)/31 (13)	62 (23) *d*; less than 3 mo	9.1 (2.3)^b^	ActiGraph GT1M or GT3X + accelerometer; MVPA, min/d, and number of MVPA bouts lasting ≥ 10 min	Objective MVPA
Muñoz‐Hernández et al. [30], Mexico	Observational cross‐sectional study with controls matched by sex and age	54/54	28 (51.9)/28 (51.9)	39 (IQR, 28–46)/39 (IQR, 28–47)	21 (10)	7.9 (IQR, 7.3–8.7)	IPAQ‐9; baseline total PA, reported as METs/wk	Total PA
Murillo et al. [27], Spain	Cross‐sectional observational study	232/248	85 (36.6)/132 (53.2)	37.6 (12.4)/36.5 (10.6)	16.0 (10.1)	7.6 (1.2)	IPAQ‐SF; total PA, MET‐min/wk, and achievement of ≥ 150 min/wk of MVPA	Total PA; meeting PA
Nunley et al. [20], US	Cross‐sectional observational cohort analysis	79/122	41 (51.9)/66 (54.1)	49.7 (7.2)/48.4 (7.4)	41 (6)	NR^c^	Modified Paffenbarger Physical Activity Questionnaire; estimated weekly PA energy expenditure, kcal/wk	Total PA
Rasmussen et al. [26], Denmark	Cross‐sectional DIAFALL study	111/328	63 (56.8)/206 (62.8)^d^	52.9 (15.3)/57.7 (15.8)	26.44 (14.2)	8.0 (1.1)^b^	Self‐reported leisure‐time PA more than twice and 30 min/wk; study‐defined active category	Meeting/active PA
Roberts et al. [23], Australia	Case‐control study with age‐ and sex‐matched controls	20/10	7 (35.0)/4 (40.0)	35 (8)/35 (9)	19 (10)	8.1 (1.5)^e^	Four‐week exercise diary; product of weekly exercise duration and intensity, MET‐h/wk	Total PA
Sorola et al. [31], Finland	Cross‐sectional matched case‐control study	15/33	8 (53.3)/14 (42.4)	32 (9)/33 (7)	15 (7)	7.5 (0.7)^f^	Self‐reported weekly frequency and duration of light‐, moderate‐, and high‐intensity PA and total PA, h/wk	Total PA
Tentolouris et al. [24], Greece	Cross‐sectional study	79/53	52 (65.8)/31 (58.5)	38.2 (12.5)/40.3 (16.7)	18.3 (9.1)	7.2 (IQR, 6.6–9.9)^b^	IPAQ long form; total PA, MET‐min/wk	Total PA
Vézina‐Im et al. [25], Canada	Cross‐sectional web‐based questionnaire study	30/97	21 (70.0)/83 (85.6)	38.6 (11.9)/30.2 (10.6)	80% had T1D for more than 10 y	NR	Godin‐shephard leisure‐time exercise questionnaire; weekly sessions of ≥ 30 min of light‐, moderate‐, and vigorous‐intensity PA	Total PA

*Note:* Data are mean (SD) unless otherwise indicated.

Abbreviations: AHA, American Heart Association; CI, confidence interval; IPAQ, International Physical Activity Questionnaire; IQR, interquartile range; MET, metabolic equivalent of task; MVPA, moderate‐to‐vigorous physical activity; NR, not reported; PA, physical activity; PAL, physical activity level; SQUASH, Short Questionnaire to Assess Health‐Enhancing Physical Activity; T1D, type 1 diabetes.

^a^ Chantelau and Wirth [16] reported 14 participants receiving multiple injections and 20 receiving continuous subcutaneous insulin infusion, each compared with matched controls. Sex totals and pooled means were calculated across these two matched comparisons. A separate pool of 53 controls was used to validate the motion metre.

^b^ Converted from IFCC units in mmol/mol to NGSP percentage using the standard conversion equation.

^c^ Current HbA1c was not reported as a baseline characteristic; the study instead used HbA1c‐months as an index of long‐term hyperglycaemic exposure.

^d^ Rasmussen et al. [26] reported 63 women as 57.7%; 63 of 111 corresponds to 56.8%.

^e^ Roberts et al. [23] reported 65 (16) mmol/mol and 8.1 (3.9)%. The percentage SD printed in the article is inconsistent with the IFCC value; conversion gives approximately 8.1 (1.5)%.

^f^ Sorola et al. [31] reported 7.5 (0.7)% in Table 1, whereas the accompanying narrative rounded the mean to 7.4%.

### 3.3 Risk of Bias Within Studies

Risk‐of‐bias assessments are presented in Supporting Information S1: Table 3. Among the 13 analytical cross‐sectional studies, participants and study settings were generally described adequately, and physical activity outcomes were usually assessed using validated questionnaires or device‐based methods. The most frequent concerns involved incomplete reporting of eligibility or diagnostic criteria, reliance on self‐reported measures, and insufficient adjustment for potential confounding in some between‐group comparisons. One study relied on self‐reported diabetes status, and a small number of studies used nonvalidated or insufficiently described physical activity measures. Among the 3 case‐control studies, matching procedures and statistical analyses were generally appropriate, although the validity of the physical activity assessment was unclear in 2 studies. No overall risk‐of‐bias score was calculated; each methodological domain was evaluated separately.

### 3.4 Results of Individual Studies and Synthesis of Results

#### 3.4.1 Total Physical Activity

Participants with type 1 diabetes had lower total physical activity levels than controls, although the magnitude of the difference was small (Hedges *g* = −0.13; 95% CI, −0.22 to −0.05; *P* = 0.001). No statistical heterogeneity was observed (*I* ^2^ = 0%; *τ* ^2^ = 0; Q(11) = 7.29; *P* = 0.775) (Figure 2).

**FIGURE 2:**
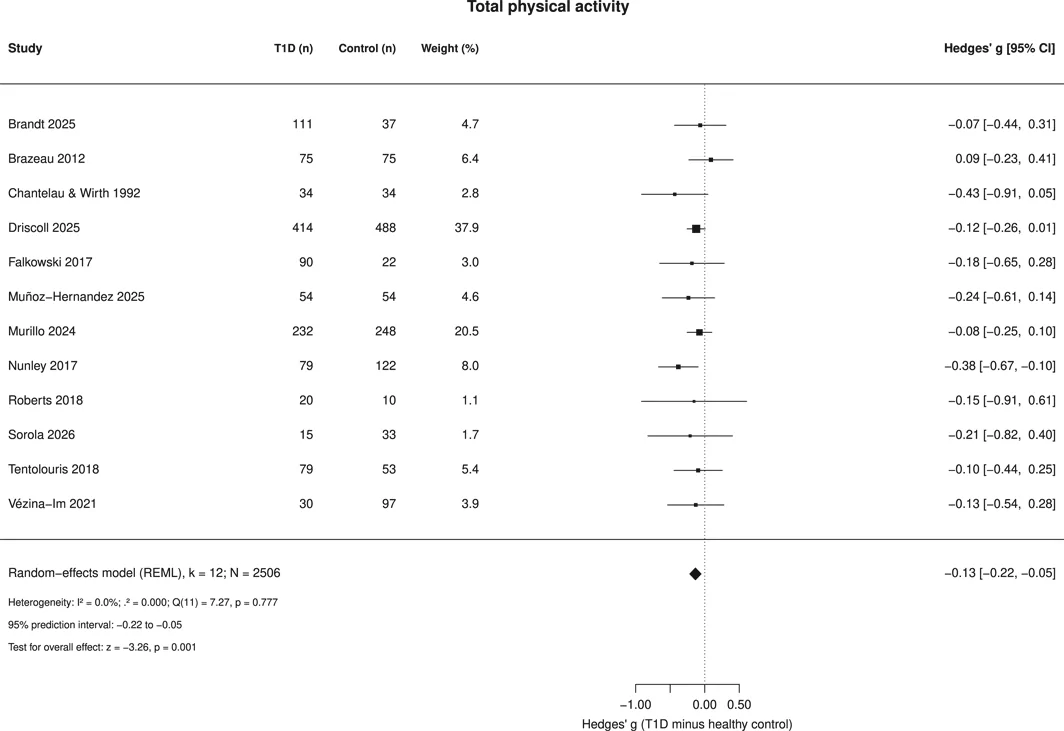
Total physical activity in adults with type 1 diabetes compared with adults without diabetes. Forest plot showing study‐specific and pooled Hedges' g values with 95% CIs. Negative values indicate lower total physical activity in adults with type 1 diabetes.

Results were consistent across sensitivity analyses. The model using the Hartung‐Knapp adjustment yielded a Hedges *g* of −0.13 (95% CI, −0.21 to −0.06; *P* = 0.002), whereas the Paule‐Mandel, DerSimonian‐Laird, and common‐effect models produced estimates that were essentially identical to the primary analysis (Supporting Information S1: Figure 1).

In leave‐one‐out analyses, pooled estimates ranged from −0.15 to −0.11, and the association remained statistically significant after each study was removed individually (Supporting Information S1: Table 4).

#### 3.4.2 Meeting a Physical Activity Criterion

Participants with type 1 diabetes had lower estimated odds of meeting the physical activity criterion, but the association was not statistically significant (OR = 0.77; 95% CI, 0.58–1.02; *p* = 0.066). Heterogeneity was moderate (*I* ^2^ = 54.1%; *τ* ^2^ = 0.044; *Q*3 = 6.06; *p* = 0.110) (Figure 3).

**FIGURE 3:**
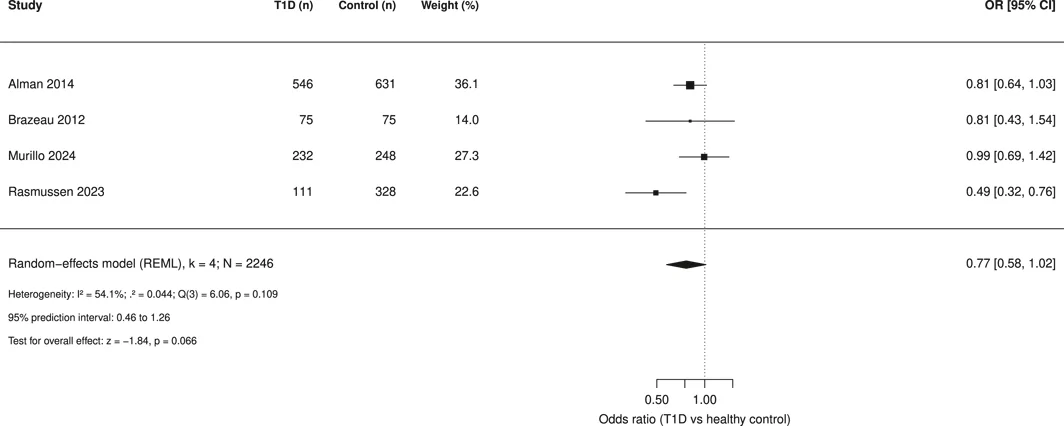
Meeting or being classified as active according to physical activity criteria in adults with type 1 diabetes compared with adults without diabetes.

With the Hartung‐Knapp adjustment, the estimate was less precise (OR = 0.77; 95% CI, 0.48–1.22; *p* = 0.163). The DerSimonian‐Laird model produced an OR of 0.77 (95% CI, 0.58–1.01; *p* = 0.057). Although the common‐effect model showed a statistically significant association (OR = 0.79; 95% CI, 0.66–0.93; *p* = 0.006), this estimate was considered secondary because the included studies used non‐equivalent definitions of physical activity and adherence (Supporting Information S1: Figure 2).

In leave‐one‐out analyses, pooled ORs ranged from 0.69 to 0.86. The association became statistically significant only after exclusion of Murillo et al. [27] (OR = 0.69; 95% CI, 0.50–0.97; *p* = 0.032); estimates remained nonsignificant after exclusion of each of the other studies (Supporting Information S1: Table 5).

### 3.5 Risk of Bias Across Studies

For the total physical activity meta‐analysis, there was no statistical evidence of small‐study effects based on either the rank correlation test (Kendall *τ* = −0.30; *p* = 0.20) or the regression test (*z* = −0.70; *p* = 0.48) (Supporting Information S1: Figure 3).

### 3.6 Additional Analysis

Figure 4 shows exploratory meta‐regression analyses examining whether the between‐group difference in total physical activity (Hedges g) was associated with mean age, mean diabetes duration, and mean HbA1c in the type 1 diabetes group. HbA1c data were available for 10 studies. Mean HbA1c was not associated with the between‐group difference in total physical activity. For each 1‐percentage‐point increase in HbA1c, the estimated change in Hedges *g* was 0.030 (95% CI, −0.294 to 0.354; *p* = 0.837).

**FIGURE 4:**
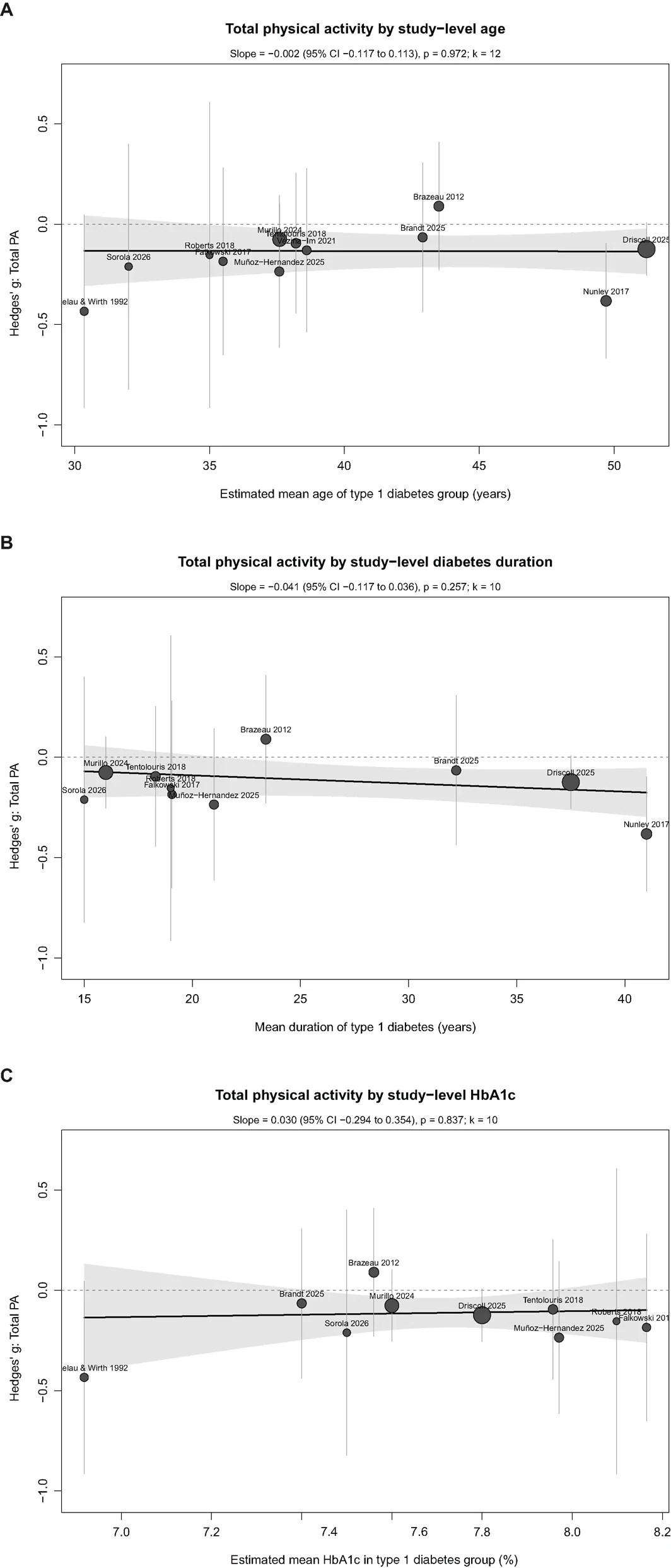
Meta‐regression analyses examining whether the between‐group difference in total physical activity (Hedges g) was associated with (A) mean age, (B) mean diabetes duration and (C) mean HbA1c in the type 1 diabetes group.

Mean diabetes duration was available for 10 studies. Each 10‐year increase in diabetes duration was associated with a change of −0.041 in Hedges *g* (95% CI, −0.117 to 0.036; *p* = 0.257).

Mean age was available for all 12 studies and was not associated with the effect size. For each 10‐year increase in age, the estimated change in Hedges *g* was −0.002 (95% CI, −0.117 to 0.113; *p* = 0.972).

## 4 Discussion

This systematic review and meta‐analysis found that adults with type 1 diabetes had lower total physical activity than adults without diabetes. The difference was small but consistent across sensitivity analyses. Adults with type 1 diabetes also had lower estimated odds of meeting a physical activity criterion, although this association was not statistically significant and showed moderate heterogeneity. Overall, the evidence suggests a modest reduction in physical activity among adults with type 1 diabetes, whereas differences in meeting specific activity thresholds remain uncertain.

The small difference in total physical activity should not necessarily be considered clinically irrelevant. Modest reductions in activity, when sustained over time, may be important in a population with an already elevated cardiovascular risk [5]. In nationwide Swedish and EURODIAB cohorts, lower physical activity was associated with higher risks of all‐cause mortality and cardiovascular disease, although some estimates were of borderline statistical significance [8, 9]. Lower physical activity may also emerge early in life, as a previous meta‐analysis found that children and adolescents with type 1 diabetes accumulated approximately 13 fewer minutes per day of moderate‐to‐vigorous physical activity and were less likely to meet physical activity recommendations than peers without diabetes [11]. Our findings suggest that these differences may persist, although to a modest extent, into adulthood.

Several diabetes‐specific factors may contribute to the lower physical activity observed in adults with type 1 diabetes. Physical activity often requires advance planning, additional glucose monitoring, and adjustments to insulin delivery or carbohydrate intake, while uncertainty about the glycaemic response and fear of hypoglycemia may discourage spontaneous or prolonged activity [1, 7, 17]. These additional self‐management demands may partly explain the lower physical activity observed in this population.

Several methodological factors may explain the small pooled difference in total physical activity. Most included studies relied on questionnaires, activity histories, or diaries, which are susceptible to recall error, social‐desirability bias, and limited precision when distinguishing activity intensity or sedentary time [18, 20, 23, 31]. Such limitations may attenuate differences between groups. Nevertheless, no statistical heterogeneity was detected in the meta‐analysis, suggesting that the direction and magnitude of the effect were broadly consistent across studies despite differences in physical activity instruments and outcome definitions. By contrast, the two additional studies using ActiGraph accelerometers reported lower device‐measured moderate‐to‐vigorous physical activity among adults with type 1 diabetes [21, 22]. However, the small number of device‐based studies and differences in monitoring protocols prevented a reliable pooled analysis. Further research using standardized accelerometer procedures is therefore needed.

Evidence regarding sedentary behaviour was limited and did not show a consistent excess among adults with type 1 diabetes. Matson et al. [22] found that adults with recently diagnosed type 1 diabetes spent less time sedentary than controls overall, although sedentary time during weekdays was similar between groups. Tentolouris et al. [24] also reported comparable self‐reported sitting time in adults with and without type 1 diabetes, while Murillo et al. [27] found a similar prevalence of sedentariness between groups (27.3% vs. 25.1%). These findings should be interpreted cautiously because sedentary behaviour was assessed using different approaches, including accelerometry, self‐reported sitting time, and categorical classifications, and the study populations differed substantially in diabetes duration and clinical characteristics. The absence of consistent between‐group differences does not diminish the clinical importance of sedentary behaviour, as prolonged occupational sitting has been associated with increased cardiovascular risk in adults with type 1 diabetes independently of leisure‐time physical activity [10]. Further studies using standardized device‐based measures are needed to determine whether total sedentary time, prolonged sedentary bouts, or patterns of interruption differ between adults with and without type 1 diabetes.

The exploratory meta‐regressions did not identify associations between the difference in total physical activity and study‐level age, diabetes duration, or HbA1c. Although HbA1c has previously been associated with cardiorespiratory fitness in type 1 diabetes [12], our results do not indicate that glycaemic control explains between‐study differences in habitual physical activity. Nevertheless, the analyses included only 10–12 studies and were based on aggregated data. They should therefore not be interpreted as evidence that these characteristics are unrelated to physical activity at the individual level.

Several limitations should be acknowledged. Most included studies were cross‐sectional, preventing causal interpretation. Physical activity instruments, recall periods, monitoring procedures, intensity thresholds, units, and outcome definitions differed substantially, and most evidence was based on self‐report. Some studies had small samples or imbalances in age and sex between groups, and adjustment for confounding was inconsistent. Importantly, none of the included studies used population‐based sampling. Instead, participants were predominantly recruited from clinical settings, convenience samples, or volunteer populations, potentially introducing selection bias and limiting the representativeness and generalisability of the findings. Data were insufficient to pool sedentary time, steps, light physical activity, or other intensity‐specific outcomes, despite their potential clinical relevance. The evidence was also concentrated in Europe, North America, and Australia, limiting generalisability to other populations. One included study was conducted in 1992 ^16^, before major advances in contemporary type 1 diabetes management and diabetes technology; however, its exclusion in the leave‐one‐out analysis did not materially alter the pooled estimate. Finally, standardized mean differences enabled the combination of different instruments but make the clinical magnitude of the pooled effect less intuitive.

## 5 Conclusion

Adults with type 1 diabetes appear to have lower total physical activity than adults without diabetes, although the evidence that they are less likely to meet a physical activity recommendation remains uncertain. Available data are insufficient to determine whether adults with type 1 diabetes accumulate more sedentary time or differ consistently in specific activity intensities. Future studies should prioritise prospective designs, standardized device‐based assessments, harmonised definitions of physical activity and sedentary behaviour, and detailed reporting of relevant diabetes‐related characteristics.

## Author Contributions

**Antonio García‐Hermoso:** conceptualization, methodology, formal analysis, investigation, data curation, visualization, writing – original draft, and project administration. **Jacinto Muñoz‐Pardeza:** investigation, data curation, validation, and writing – review and editing. **Ignacio Hormazábal‐Aguayo:** interpretation of results and writing – review and editing. **Yasmin Ezzatvar:** conceptualization, methodology, investigation, data curation, validation, and writing – review and editing. All authors read and approved the final manuscript and agree to be accountable for all aspects of the work.

## Funding

No external funding was received for this study. Open access funding provided by Universidad Publica de Navarra.

## Conflicts of Interest

The authors declare no conflicts of interest.

## Supporting information


Supporting Information S1

## Data Availability

The data that support the findings of this study are available from the corresponding author upon reasonable request.
