# Supplementary material for: Adults With Type 1 Diabetes Are Less Physically Active Than Adults Without Diabetes: A Systematic Review and Meta‐Analysis
Source: Diabetes Metab Res Rev. 2026 Nov 6;42(8):e70250. doi: 10.1002/dmrr.70250 (PMC13741197; doi:10.1002/dmrr.70250)
Supplement: Supplementary file 1 [file DMRR-42-e70250-s001.docx]

**Supplementary material**

Adults with type 1 diabetes are less physically active than adults without diabetes: a systematic review and meta-analysis

**Supplementary Table 1. Electronic search strategies used in PubMed, Embase, and SPORTDiscus.**

**PubMed n = 2231**

( "type 1 diabetes mellitus"[MeSH Terms] OR "type 1 diabetes" OR "type 1 diabetic" OR T1D OR T1DM ) AND ( "Motor Activity"[MeSH Terms] OR "Exercise"[MeSH Terms] OR "Sedentary Behavior"[MeSH Terms] OR "physical activity" OR "leisure time physical activity" OR "moderate-to-vigorous physical activity" OR MVPA OR exercise OR acceleromet* OR actigraph* OR pedomet* OR "step count" OR "daily steps" OR "energy expenditure") AND ( healthy control* OR control* OR non-diabetic OR nondiabetic )

**SportDiscus n = 92**

(	MH "Diabetes Mellitus, Insulin-Dependent"	OR TI (	"type 1 diabetes mellitus"	OR "type 1 diabetes"	OR "type 1 diabetic*"	OR T1D	OR T1DM	OR IDDM	OR "insulin-dependent diabetes"	OR "insulin dependent diabetes"	)	OR AB (	"type 1 diabetes mellitus"	OR "type 1 diabetes"	OR "type 1 diabetic*"	OR T1D	OR T1DM	OR IDDM	OR "insulin-dependent diabetes"	OR "insulin dependent diabetes"	) ) AND (	MH "Physical Activity"	OR MH "Exercise"	OR MH "Motor Activity"	OR MH "Sedentary Behavior"	OR MH "Life Style, Sedentary"	OR TI (	"physical activit*"	OR "leisure time physical activit*"	OR "moderate-to-vigorous physical activit*"	OR "moderate to vigorous physical activit*"	OR MVPA	OR exercis*	OR sedentar*	OR acceleromet*	OR actigraph*	OR pedomet*	OR "step count*"	OR "daily step*"	OR "energy expenditure"	)	OR AB (	"physical activit*"	OR "leisure time physical activit*"	OR "moderate-to-vigorous physical activit*"	OR "moderate to vigorous physical activit*"	OR MVPA	OR exercis*	OR sedentar*	OR acceleromet*	OR actigraph*	OR pedomet*	OR "step count*"	OR "daily step*"	OR "energy expenditure"	) ) AND (	TI (	"healthy control*"	OR "healthy participant*"	OR "healthy subject*"	OR "control group*"	OR (control* N3 (group* OR participant* OR subject* OR individual* OR person* OR people))	OR non-diabet*	OR nondiabet*	OR "non diabet*"	)	OR AB (	"healthy control*"	OR "healthy participant*"	OR "healthy subject*"	OR "control group*"	OR (control* N3 (group* OR participant* OR subject* OR individual* OR person* OR people))	OR non-diabet*	OR nondiabet*	OR "non diabet*"	) )

**Embase n = 2593**

('type 1 diabetes mellitus'/exp OR 'type 1 diabetes':ti,ab,kw OR 'type 1 diabetic':ti,ab,kw OR 'type 1 diabetics':ti,ab,kw OR t1d:ti,ab,kw OR t1dm:ti,ab,kw) AND ('physical activity'/exp OR 'sedentary behavior'/exp OR ((physical NEAR/2 activ*):ti,ab,kw) OR ((leisure NEAR/3 activ*):ti,ab,kw) OR ((moderate NEAR/3 vigorous NEAR/3 activ*):ti,ab,kw) OR mvpa:ti,ab,kw OR sedentary:ti,ab,kw OR ((sitting NEAR/1 time):ti,ab,kw) OR ((screen NEAR/1 time):ti,ab,kw) OR acceleromet*:ti,ab,kw OR actigraph*:ti,ab,kw OR pedomet*:ti,ab,kw OR ((step NEAR/2 count*):ti,ab,kw) OR ((steps NEAR/2 day):ti,ab,kw) OR ((energy NEAR/1 expenditure):ti,ab,kw) OR (((cardiorespiratory OR aerobic OR physical) NEAR/2 (fitness OR capacity)):ti,ab,kw) OR vo2max:ti,ab,kw OR vo2peak:ti,ab,kw) AND (control*:ti,ab,kw OR healthy:ti,ab,kw OR nondiabet*:ti,ab,kw OR ((non NEAR/1 diabet*):ti,ab,kw) OR compar*:ti,ab,kw)

**Supplementary Table 2. Studies excluded after full-text assessment and reasons for exclusion from the primary quantitative syntheses.**

When more than one reason applied, the principal reason for exclusion was reported. T1D indicates type 1 diabetes; CACTI, Coronary Artery Calcification in Type 1 Diabetes; MVPA, moderate-to-vigorous physical activity; PA, physical activity; PASE, Physical Activity Scale for the Elderly.

| **Reference** | **Reason for exclusion from primary quantitative synthesis** |
| --- | --- |
| **Basu et al. (2026)** | Overlapping CACTI cohort; physical activity was included as an adjustment variable rather than reported as an independent between-group physical activity outcome |
| **Benbassat et al. (2001)** | No healthy control group and no eligible habitual physical activity or sedentary-behaviour outcome |
| **Brugnara et al. (2012)** | Healthy controls were selected or matched according to exercise status, cardiorespiratory fitness, and body composition |
| **Çelik et al. (2023)** | Physical activity was assessed during the COVID-19 pandemic restriction period |
| **Dial et al. (2021)** | Healthy controls were matched according to self-reported physical activity level |
| **Eckstein et al. (2021)** | No eligible habitual physical activity or sedentary-behaviour outcome; pooled analysis focused on physiological responses during cardiopulmonary exercise testing |
| **Ewers et al. (2019)** | Physical activity was assessed using different methods in the T1D and general-population groups, preventing a directly comparable quantitative estimate |
| **Finn et al. (2021/2022)** | No healthy control group available for comparison |
| **Goulding et al. (2020)** | Participants with T1D were selected as recreationally active, and healthy controls were selected to have a similar physical activity status |
| **Grabia et al. (2021)** | Physical activity was assessed during the COVID-19 pandemic restriction period |
| **Greco et al. (2026)** | Healthy controls were matched according to self-reported physical activity level |
| **Hägglund et al. (2012)** | Healthy controls were matched according to leisure-time physical activity |
| **Hyrylä et al. (2022)** | No eligible habitual physical activity or sedentary-behaviour outcome; study focused on ventilatory responses during cardiopulmonary exercise testing |
| **Jabbour et al. (2022)** | No healthy control group and physical activity was assessed during the COVID-19 period |
| **Jlali et al. (2023)** | Healthy controls were matched according to physical activity level and body composition |
| **Komatsu et al. (2010)** | Participants were selected and stratified according to athletic or programmed-exercise status |
| **Koponen et al. (2013)** | Healthy controls were matched according to leisure-time physical activity |
| **Kremser et al. (1988)** | No eligible habitual physical activity or sedentary-behaviour outcome; study assessed physiological responses during exercise |
| **Kujath et al. (2015)** | Physical activity was reported only using study-specific vigorous-activity categories that were not compatible with the predefined Total PA, MVPA, sedentary-time, or guideline-adherence syntheses |
| **Kulecki et al. (2025)** | Participants were selected because of regular endurance exercise or athletic participation |
| **Lee et al. (2016)** | Healthy controls were matched according to physical activity or exercise history |
| **Monaco et al. (2021)** | Healthy controls were matched according to physical activity level |
| **Moser et al. (2018)** | No healthy control group available for comparison |
| **Nugent et al. (1997)** | No eligible habitual physical activity or sedentary-behaviour outcome; study assessed physiological and metabolic responses during exercise |
| **Peltonen et al. (2012)** | Healthy controls were matched according to self-reported leisure-time physical activity |
| **Ramires et al. (1993)** | No eligible habitual physical activity or sedentary-behaviour outcome; study assessed exercise tolerance and acute metabolic responses |
| **Richardson et al. (2023)** | Overlapping CACTI cohort and repeated self-reported physical activity data; Alman et al. (2014), Driscoll et al. (2025), and Keshawarz et al. (2018) were retained in their respective outcome-specific analyses to avoid double-counting participants |
| **Rissanen et al. (2015)** | Healthy controls were matched according to self-reported leisure-time physical activity |
| **Rissanen et al. (2018)** | Exercise-training intervention with no eligible independent baseline comparison of habitual physical activity |
| **Roszkowski et al. (2019)** | The T1D sample was restricted to young insulin-pump users and showed unusually high physical activity levels, resulting in a high risk of selection bias and limited comparability with the control group |
| **Sarısakaloğlu et al. (2026)** | Physical activity was assessed during a period affected by COVID-19 pandemic restrictions |
| **Stewart et al. (2017)** | Participants with T1D were selected for high physical fitness, and healthy controls were matched according to exercise habits and cardiorespiratory fitness |
| **Tagougui et al. (2015)** | Healthy controls were matched according to physical activity level and body composition |
| **Tan et al. (2022)** | Healthy controls were matched according to physical activity level |
| **Turčinović et al. (2023)** | No healthy control group available for comparison |
| **Turinese et al. (2017)** | Healthy controls were matched according to physical activity level |
| **Turner et al. (2022)** | Healthy controls were matched according to PASE physical activity score |
| **Veves et al. (1997)** | Participants were selected according to regular exercise participation, and comparison groups were constructed to have comparable weekly physical activity energy expenditure |
| **Waclawovsky et al. (2016)** | No eligible habitual physical activity or sedentary-behaviour outcome; study evaluated acute responses to experimental exercise sessions |
| **Wanke et al. (1992)** | No eligible habitual physical activity or sedentary-behaviour outcome; study assessed exercise capacity and pulmonary gas exchange |
| **Wilson et al. (2017)** | Healthy controls were matched according to self-reported exercise participation |
| **Żebrowska et al. (2018)** | No eligible habitual physical activity or sedentary-behaviour outcome; study evaluated acute physiological responses to exercise and hypoxia |
| **Zhao et al. (2008)** | Type 1 and type 2 diabetes were combined, and T1D-specific physical activity results were not reported separately |

**Supplementary Table 3. Risk-of-bias assessment of the included studies using the Joanna Briggs Institute critical appraisal checklists.**

**Panel A. JBI Critical Appraisal Checklist for Analytical Cross-Sectional Studies**

| **Study** | **Q1** | **Q2** | **Q3** | **Q4** | **Q5** | **Q6** | **Q7** | **Q8** |
| --- | --- | --- | --- | --- | --- | --- | --- | --- |
| Alman et al., 2014 | U | Y | U | U | Y | Y | Y | Y |
| Brazeau et al., 2012 | Y | Y | Y | Y | Y | Y | Y | Y |
| Falkowski et al., 2017 | U | Y | U | U | Y | N | Y | N |
| Nunley et al., 2017 | Y | Y | Y | U | Y | N | Y | U |
| Keshawarz et al., 2018 | U | Y | U | U | Y | Y | Y | Y |
| Matson et al., 2018 | Y | Y | Y | Y | Y | Y | Y | Y |
| Tentolouris et al., 2018 | Y | Y | Y | Y | Y | Y | Y | Y |
| Vézina-Im et al., 2021 | Y | Y | U | N | Y | U | Y | Y |
| Rasmussen et al., 2023 | Y | Y | Y | Y | Y | N | U | N |
| Murillo et al., 2024 | Y | Y | U | U | Y | Y | Y | Y |
| Brandt et al., 2025 | Y | Y | Y | Y | Y | Y | Y | Y |
| Driscoll et al., 2025 | U | Y | Y | Y | Y | U | Y | U |
| Muñoz-Hernandez et al., 2025 | Y | Y | Y | U | Y | Y | Y | Y |

**Panel A checklist items**

Q1. Were the criteria for inclusion in the sample clearly defined?

Q2. Were the study subjects and the setting described in detail?

Q3. Was the exposure measured in a valid and reliable way?

Q4. Were objective, standard criteria used for measurement of the condition?

Q5. Were confounding factors identified?

Q6. Were strategies to deal with confounding factors stated?

Q7. Were the outcomes measured in a valid and reliable way?

Q8. Was appropriate statistical analysis used?

**Panel B. JBI Critical Appraisal Checklist for Case-Control Studies**

| **Study** | **Q1** | **Q2** | **Q3** | **Q4** | **Q5** | **Q6** | **Q7** | **Q8** | **Q9** | **Q10** |
| --- | --- | --- | --- | --- | --- | --- | --- | --- | --- | --- |
| Chantelau and Wirth, 1992 | Y | Y | U | Y | Y | Y | Y | Y | Y | Y |
| Roberts et al., 2018 | Y | Y | Y | U | Y | Y | Y | Y | Y | Y |
| Sorola et al., 2026 | Y | Y | Y | U | Y | Y | Y | Y | Y | Y |

**Abbreviations:** Y, yes; N, no; U, unclear or insufficiently reported.

**Interpretation note:** For the analytical cross-sectional checklist, type 1 diabetes status was considered the exposure/condition and physical activity or sedentary behavior the outcome. No composite JBI score or overall risk category was calculated.

**Panel B checklist items**

Q1. Were the groups comparable other than the presence of disease in cases or the absence of disease in controls?

Q2. Were cases and controls matched appropriately?

Q3. Were the same criteria used for identification of cases and controls?

Q4. Was exposure measured in a standard, valid, and reliable way?

Q5. Was exposure measured in the same way for cases and controls?

Q6. Were confounding factors identified?

Q7. Were strategies to deal with confounding factors stated?

Q8. Were outcomes assessed in a standard, valid, and reliable way for cases and controls?

Q9. Was the exposure period of interest long enough to be meaningful?

Q10. Was appropriate statistical analysis used?

**Supplementary Figure 1. Sensitivity analyses using alternative meta-analytic models for total physical activity.**

The primary random-effects model using restricted maximum likelihood estimation was compared with random-effects models using the Hartung-Knapp adjustment, Paule-Mandel estimation, and DerSimonian-Laird estimation, and with a common-effect inverse-variance model. Effect estimates are expressed as Hedges g with 95% CIs. Negative values indicate lower total physical activity in participants with type 1 diabetes than in controls. CI indicates confidence interval; REML, restricted maximum likelihood.

**Supplementary Table 4. Leave-one-out sensitivity analysis for total physical activity.**

Each row presents the pooled Hedges g after sequential exclusion of the indicated study. Negative values indicate lower total physical activity in participants with type 1 diabetes than in controls. I² indicates the proportion of variability attributable to between-study heterogeneity; SE, standard error; τ², estimated between-study variance.

| Removed_study | Hedges_g | se | pval | tau^2^ | I^2^ |
| --- | --- | --- | --- | --- | --- |
| Brandt 2025 | -0.138 | 0.042 | 0.001 | 0 | 0.0 |
| Brazeau 2012 | -0.15 | 0.043 | 0.0 | 0 | 0.0 |
| Chantelau & Wirth 1992 | -0.126 | 0.042 | 0.003 | 0 | 0.0 |
| Driscoll 2025 | -0.141 | 0.052 | 0.007 | 0 | 0.0 |
| Falkowski 2017 | -0.133 | 0.042 | 0.001 | 0 | 0.0 |
| Muñoz-Hernandez 2025 | -0.13 | 0.042 | 0.002 | 0 | 0.0 |
| Murillo 2024 | -0.15 | 0.046 | 0.001 | 0 | 0.0 |
| Nunley 2017 | -0.113 | 0.043 | 0.009 | 0 | 0.0 |
| Roberts 2018 | -0.134 | 0.041 | 0.001 | 0 | 0.0 |
| Sorola 2026 | -0.133 | 0.042 | 0.001 | 0 | 0.0 |
| Tentolouris 2018 | -0.137 | 0.042 | 0.001 | 0 | 0.0 |
| Vézina-Im 2021 | -0.135 | 0.042 | 0.001 | 0 | 0.0 |

**Supplementary Figure 2. Sensitivity analyses using alternative meta-analytic models for meeting a physical activity recommendation or study-defined active criterion.**
The primary random-effects model using restricted maximum likelihood estimation was compared with random-effects models using the Hartung-Knapp adjustment, Paule-Mandel estimation, and DerSimonian-Laird estimation, and with a common-effect inverse-variance model. Effect estimates are expressed as odds ratios with 95% CIs. An odds ratio below 1 indicates lower odds of meeting the physical activity criterion among participants with type 1 diabetes. CI indicates confidence interval; OR, odds ratio; REML, restricted maximum likelihood.

**Supplementary Table 5. Leave-one-out sensitivity analysis for meeting a physical activity recommendation or study-defined active criterion.**
Each row presents the pooled odds ratio after sequential exclusion of the indicated study. An odds ratio below 1 indicates lower odds of meeting the physical activity criterion among participants with type 1 diabetes. I² indicates the proportion of variability attributable to between-study heterogeneity; OR, odds ratio; SE, standard error; τ², estimated between-study variance.

| Removed_study | OR | se | pval | tau^2^ | I^2^ |
| --- | --- | --- | --- | --- | --- |
| Alman 2014 | 0.74 | 0.23 | 0.182 | 0.10 | 64.03 |
| Brazeau 2012 | 0.75 | 0.19 | 0.128 | 0.07 | 71.61 |
| Murillo 2024 | 0.69 | 0.17 | 0.032 | 0.04 | 49.73 |
| Rasmussen 2023 | 0.86 | 0.10 | 0.109 | 0.00 | 0.00 |

**Supplementary Figure 3. Funnel plot for the meta-analysis of total physical activity.**

The horizontal axis represents the observed standardized mean difference (Hedges g), and the vertical axis represents its standard error. The vertical dashed line indicates the pooled effect estimate, and the diagonal dashed lines represent the approximate 95% confidence limits. Visual inspection did not suggest substantial funnel plot asymmetry. Formal tests showed no evidence of small-study effects (Begg rank correlation test, Kendall τ = −0.30; P = 0.20; Egger-type regression test, z = −0.70; P = 0.48).
